# Separation of Boron and Arsenic from Geothermal Water with Novel Gel-Type Chelating Ion Exchange Resins: Batch and Column Sorption-Elution Studies

**DOI:** 10.3390/molecules28237708

**Published:** 2023-11-22

**Authors:** Esra Altıok, Fatma Şen, Joanna Wolska, Piotr Cyganowski, Marek Bryjak, Nalan Kabay, Müşerref Arda, Mithat Yüksel

**Affiliations:** 1 Chemical Engineering Department, Faculty of Engineering, Ege University, Izmir 35100, Turkey; 2 Division of Material Science and Engineering, Graduate School of Natural and Applied Science, Ege University, Izmir 35100, Turkey; 3 Department of Process Engineering and Technology of Polymer and Carbon Materials, Wroclaw University of Science and Technology, 50-370 Wroclaw, Polandpiotr.cyganowski@pwr.edu.pl (P.C.); 4 Department of Chemistry, Faculty of Science, Ege University, Izmir 35100, Turkey

**Keywords:** arsenic, boron, chelating ion exchange resin, geothermal water, ion exchange

## Abstract

In this research, the removal of boron and arsenic from geothermal water was examined by using novel *N*-methyl-d-glucamine functionalized gel-like resins (abbreviated as 1JW and 2JW) synthesized by the membrane emulsification method. The outcomes were compared with those of commercially available boron selective chelating ion exchange resin (Diaion CRB 05). According to the results obtained with the novel resins, it was possible to reduce both boron and arsenic concentrations in geothermal water by using these novel gel-like chelating resins below their permissible levels for agricultural irrigation (<1 mg B/L) and drinking water (<0.01 mg As/L) by using the batch method. The optimum resin concentration required for almost complete boron removal (more than 95%) with the two chelating resins was determined to be 2 g/L. The novel gel-like chelating resins 1JW and 2JW achieved 94% of arsenic removal by using the resin concentration of 8 g/L, while the required resin concentration was 32 g/L for 94% of arsenic removal using commercially available Diaion CRB05 resin. In addition, the column performance characteristics of the novel chelating resins for the separation of boron were studied, and the results were compared to those obtained with Diaion CRB05. According to the column data obtained, the total resin capacities of the Diaion CRB05, 1JW, and 2JW resins were calculated as 6.29, 5.08, and 4.64 mg B/mL-resin, respectively.

## 1. Introduction

Throughout history, people have actively sought out appropriate water supply systems in order to fulfill their needs. This is the reason that many civilizations have mostly developed and expanded in close proximity to substantial water resources [1]. However, the uneven distribution of water resources throughout the world is an important problem. Although water is considered one of the most essential resources on the planet, it is still constantly threatened by climate change and consequent droughts, as well as rapid population increase and waste. These causes contribute to water contamination, limited access to clean and sanitary water, and the threat to natural water bodies [2,3,4]. The motivation for water reuse has expanded beyond just social obligation to include a broader range of factors aimed at safeguarding this important resource [5].

Geothermal water is used in direct-use and indirect applications, such as for power generation, chemical production, agriculture, fish farming, balneotherapy, and thermal tourism [6,7,8]. When utilizing geothermal water utilization for any specific purpose, an enormous amount of water is extracted from geothermal aquifers. Therefore, re-injection of geothermal fluids following their utilization into reservoirs is necessary for the long-term operation of geothermal power facilities. Due to the damage caused to the environment because of their content, geothermal fluids should be treated with proper treatment methods. Consequently, it is essential to subject geothermal fluids that have been extracted to a processing stage prior to their discharge into the surface environment or aquifer in order to ensure their sustainable disposal. This strategy represents the sole approach by which this challenge may be surmounted [9]. Additionally, the use of geothermal water, particularly irrigation water, may be an alternative approach to the water scarcity problem. However, in agricultural usage, in other words, for irrigation, it is essential to track the chemical composition of geothermal water to avoid its adverse effects. Because plant growth is affected by the chemical contents of irrigation water directly, it is not easy to use geothermal water directly for irrigation due to its salinity and the content of some harmful components [10,11].

Temperature, regional geology, the length of time the hydrothermal system has existed, and fluid source or mixing (such as seawater) influence the proportion and type of dissolved chemical species in geothermal liquids. But some components have sufficiently harmful effects, even in small amounts. It is stated that the concentrations of boron and arsenic in geothermal water are much higher than the permissible levels for drinking water. In 2011, the WHO determined the highest allowed boron level in drinkable water to be 2.4 mg/L. However, for irrigation water, this value is still 1 mg/L. The WHO provisional guideline indicated the acceptable level of arsenic in drinking water as 0.01 mg/L. In light of this fact, geothermal water that needs to be utilized or disposed of should first be treated in order to produce water that is free of potentially harmful contaminants like arsenic and boron [12]. In addition, water resources that are used for drinking and irrigation, such as surface waters and groundwater in the vicinity of geothermal facilities, have become polluted with a considerable amount of arsenic and other toxic elements as a result of the wastewater discharged from geothermal plants [13].

By using traditional treatment methods such as precipitation, coagulation, adsorption, electrocoagulation, flocculation, sedimentation, ion exchange, and membrane processes, boron as well as arsenic could be eliminated from aqueous solutions, seawater, or geothermal water. Among all of these techniques, the main disadvantage of using adsorption and ion exchange as water treatment techniques is the need for chemical agents for regeneration. Even so, adsorption and ion exchange procedures, on the contrary side, are promoted as the most advantageous processes because of the potentially serious disadvantages of the other methods [14].

The *N*-methyl-d-glucamine (NMDG) group’s capabilities, which include a remarkable selectivity for boron, have received significant attention in the field of research. Boron is captured by the NMDG functional groups of the chelating resin by means of covalent bonding and then forms a coordination complex [15]. Chelating resins containing ligands with three or more hydroxyl groups in the cis position have been stated to show a significant level of selectivity to boron [16,17].

There are plenty of alternatives for arsenic removal from aqueous solutions, including oxidation, precipitation, coagulation, adsorption, ion-exchange, and membranes filtration [18]. Among them, ion exchange is mostly used. In the literature, strong base anion exchange resins are mentioned to be effective in the removal of arsenic [19,20]. Remarkable results were obtained by employing ion-exchange resins with NMDG for arsenic sorption from an aqueous solution [20,21]. It has been shown that under acidic conditions (pH 4–6), ion exchange resins containing NMDG ligands increased removal of arsenate [21]. It was stated that the resin with NMDG had the ability for selective removal of arsenate from groundwater [22].

In this study, the gel-like novel chelating resins containing *N*-methyl-d-glucamine groups were synthesized by using the membrane emulsification method, and their ability to remove boron and arsenic from geothermal water simultaneously was investigated by using the batch method. The outcomes were compared to a commercially available glucamine-type chelating resin (Diaion CRB 05). In addition, the functionality of the resins for boron removal from the geothermal water were examined by using continuous column studies to obtain the total capacities of the resins.

## 2. Results and Discussion

### 2.1. Batch Sorption Studies

**Figure 1:**
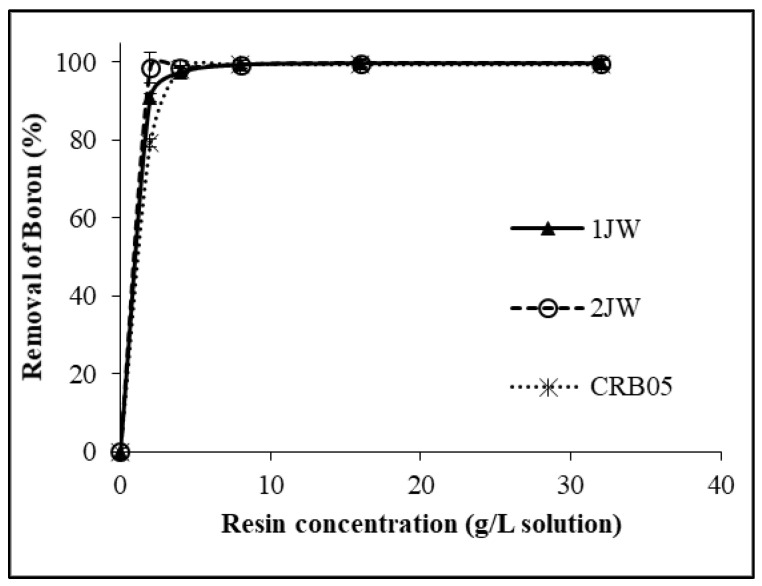
Boron removal from GW with 1JW, 2JW, and Diaion CRB 05 resins (Feed B concentration: 10.06 mg/L; 25 °C).

Figure 1 illustrates the impact of resin concentration on the removal of boron from geothermal water (GW) using 1JW, 2JW, and Diaion CRB 05. The boron concentrations remaining in the solution after adsorption are given in Table 1. The data suggest that there is a positive correlation between resin concentration and the removal of boron. Optimum resin concentrations were determined according to permissible levels for boron in the irrigation water as 1 mg/L. Hence, the most suitable concentrations of resin for the purpose of removing boron from geothermal water with the 1JW, 2JW and Diaion CRB05 resins were 2 g/L (with 91.0% of boron removal), 2 g/L (with 98.6% of boron removal) and 4 g/L (98.2% of boron removal), respectively. These findings demonstrated that when the geothermal water was used, the 1JW and 2JW resins were more effective in removing boron than Diaion CRB05 resin. It was reported that when a model boric acid solution (MBAS) containing 10 mg/L boron was used as the feed solution, 2 g/L resin amount was the optimum amount to lower the boron concentration in the solution less than 1 mg/L [23]. It can be deduced from these two studies that the amount of resin employed in the MBAS was insufficient to eliminate boron from the geothermal water due to the interference of other ions in the geothermal water.

**Figure 2:**
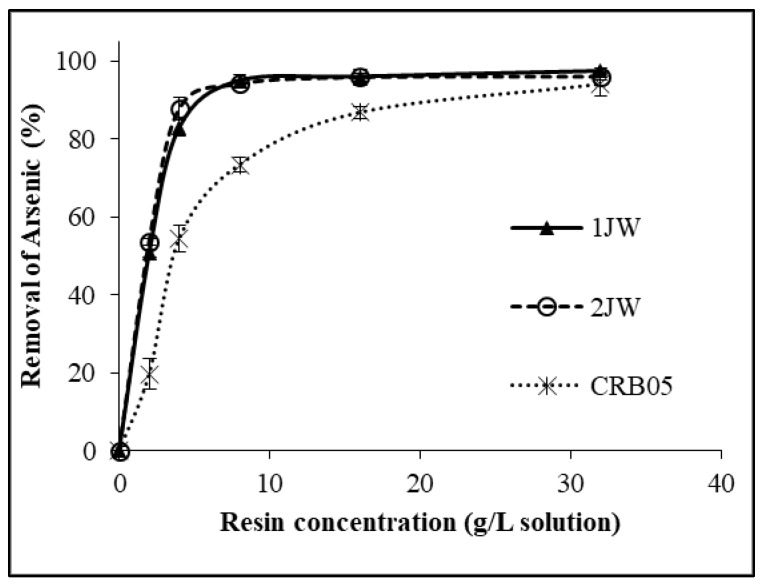
Arsenic removal from GW with 1JW, 2JW, and Diaion CRB 05 resins (Feed As concentration: 0.160 mg/L; 25 °C).

Simultaneous removal of arsenic from the geothermal water with the same resins was also investigated. As depicted in Figure 2, just like for boron removal, the percentage of arsenic removal exhibited a positive correlation with the resin concentration, wherein an increase in the resin concentration led to an improvement in the percentage of arsenic removal. However, optimum resin concentrations for arsenic removals were higher than those obtained for boron removals. The arsenic concentrations remaining in the solution after adsorption are given in Table 2. The optimum amount of resin to reduce the arsenic concentration below 0.01 mg/L was determined for each resin. At the resin concentration of 8 g/L, the 1JW and 2JW resins had arsenic removal efficiencies of 95% and 94%, respectively. For Diaion CRB 05, 90% of arsenic removal was obtained when the resin concentration was as high as 32 g/L. The novel gel-like resins (1JW and 2JW) could be considered potential resins for the simultaneous removal of boron and arsenic from geothermal water.

Table 3 presents the obtained removal of arsenic and boron and compares these results with other investigations in similar studies. It appears that boron removal using the 1JW and 2JW resins is more effective than competitors regarding the concentration of boron in the feed solution and the quantity of resin required to remove it from the solution. Comparing the performance of the commercial resin Diaion CRB05 to those mentioned in the published sources, this investigation shows that a comparable level of removal is also obtained. The 1JW and 2JW resins exhibit favorable outcomes when the data are considered in terms of arsenic removal. Furthermore, the simultaneous removal of boron and arsenic distinguishes the 1JW and 2JW resins.

### 2.2. Kinetics Studies

**Figure 3:**
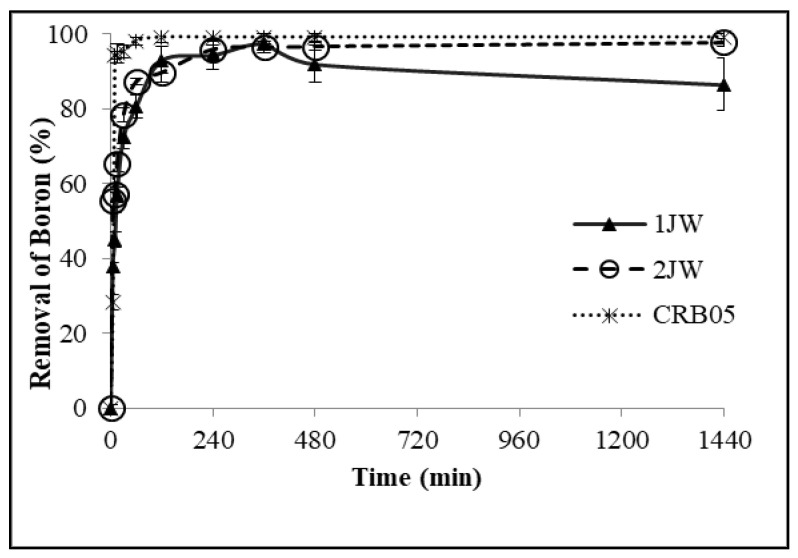
Boron removal vs. time plots for 1JW, 2JW, and Diaion CRB 05 resin (Feed B concentration: 10.06 mg/L; 25 °C).

Figure 3 displays graphs illustrating the relationship between boron removal and time for all three resins. The boron concentrations remaining in the solution after sorption are given in Table 4. Based on the results obtained from the kinetic test, it was observed that a boron removal efficiency of 94% was attained during a duration of 120 min, with a resin concentration of 4 g/L. In the context of the 2JW resin, a comparable level of uptake was obtained with a resin concentration of 2 g/L. After 10 min, the Diaion CRB05 resin exhibited a boron removal of 94% when it was used at a concentration of 4 g/L.

To determine the rate-controlling steps, the kinetic data were evaluated in spots of diffusional and reactional kinetics models [28] (Table 5).

The R^2^ values obtained from the linearized plots were recorded and shown in Table 6. In the context of traditional kinetic modeling, it can be seen that all resins conform to the pseudo-second-order kinetic model. Based on the infinite solution volume kinetic model, it can be inferred that particle diffusion was the governing factor for the reaction rate in all three resins. However, in the case of both the 1JW and 2JW resins, the reaction rate was mostly determined by a chemical reaction, as shown by the unreacted core model. In the context of Diaion CRB 05, it is noteworthy that the liquid film played a crucial role as the deciding factor for the pace of the process.

### 2.3. Batch Elution Tests

The elution efficiencies of boron from chelating resins are summarized in Table 7. By using 0.5 mol/L of H_2_SO_4_, the percent elution of boron was more than 90% for the 2JW and Diaion CRB05 resins, while it was only 74.1% for the 1JW resin. As summarized in Table 7, when the concentration of H_2_SO_4_ was raised to 1 mol/L, the elution of boron was more than 99% for the 1JW resin.

### 2.4. Column-Mode Sorption and Elution Studies

**Figure 4:**
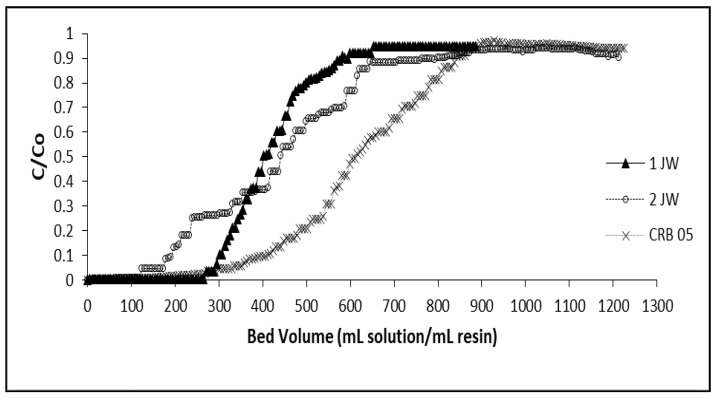
Boron concentration change vs. time plots for 1JW, 2JW, and Diaion CRB 05 resin (Feed B concentration: 10.06 mg/L; Room condition).

Column-mode sorption and elution tests for removal of boron from the geothermal water with the chelating resins were carried out, and the results obtained were compared. Breakthrough profiles of boron for the 1JW, 2JW, and Diaion CRB 05 resins are depicted in Figure 4. The closest point to the permissible concentration of boron in water used for irrigation, 1 mg/L, was accepted as the breakthrough point. The overall results of column-mode studies are summarized in Table 8. In terms of the total ion exchange capacity, the best performance was exhibited by the Diaion CRB05 resin with a total capacity of 6.29 mg B/mL-resin and a breakthrough capacity of 3.98 mg B/mL-resin. The 1JW resin followed the Diaion CRB05 resin according to its total ion exchange capacity (5.08 mg B/mL-resin) and breakthrough capacity (3.52 mg B/mL-resin). The differences in the column performances of the chelating resins are due to the differences in their polymer matrix structure and their water contents. The 2JW resin was synthesized from poly(VBC-co-DVB) copolymer, while the 1JW resin was obtained from poly(VBC-S-DVB) terpolymer. The matrix of the Diaion CRB05 resin is poly(S-co-DVB) copolymer. The water content of the 1JW and Diaion CRB05 resins are quite similar (42–43%), while the 2JW resin has a water content of 78%.

**Figure 5:**
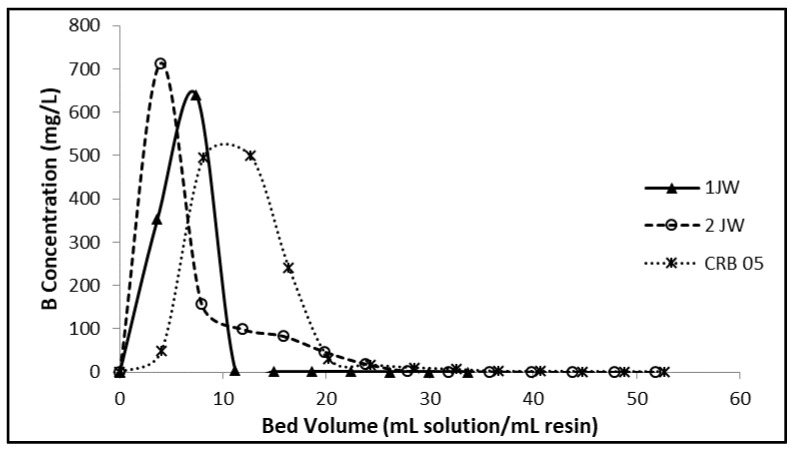
Elution curves of boron (Room condition).

The elution curves of boron from the 1JW, 2JW and Diaion CRB 05 resins are illustrated in Figure 5. The 2JW resin exhibited better elution performance (95.7%) for boron than both the Diaion CRB05 and 1JW resins.

## 3. Materials and Methods

### 3.1. Materials

After being synthesized with the membrane emulsification method, the gel-type chelating ion exchange resins (1JW and 2JW) were then subjected to suspension polymerization to complete the synthesis process. For preparation of polymer matrices, vinylbenzyl chloride (VBC), styrene (S), and divinylbenzene (DVB) were used. The polymer matrix of 1JW contained VBC/S/DVB (VBC:S = 3:1) while that of 2JW was synthesized from VBC/DVB monomers. For microsphere production, Micropore Ltd. membrane emulsification system was employed with a metal membrane having 5 μm of pores size. The process of functionalization was conducted with 1:1 (volume:volume) of dioxane:water solution with a 10 times of molar excess of *N*-methyl-d-glucamine in proportion to the chloride concentration to attach NMDG functional groups [23]. Details of the preparation protocol can be found in the literature [24]. The boron selective ion exchange resin, Diaion CRB 05, was kindly donated by Mitsubishi Chemical Co., Tokyo, Japan. The characteristics of the novel gel-type resins (1JW and 2JW) and the Diaion CRB 05 resin are presented in Table 9 and Table 10, correspondingly.

The geothermal water used for the adsorption research was sourced from Izmir Geothermal Company, located in Turkey. Table 11 presents the chemical composition of the geothermal water (GW).

### 3.2. Methods

#### 3.2.1. Batch-Mode Adsorption Tests

In batch tests, 0.01, 0.05, 0.1, 0.2, 0.4, and 0.8 g of chelating resins were brought into contact with 25 mL of geothermal water and continuously mixed for 24 h at 25 °C. Optimum resin concentrations were determined by analyzing samples for boron and arsenic in the supernatant.

#### 3.2.2. Kinetic Tests

Kinetic studies, by using the optimal amounts of resins as determined in the batch adsorption tests, were executed with the purpose of evaluating the adsorption kinetics of the resins. In order to do this, every resin was exposed to 300 mL of geothermal water and agitated using mechanical stirring at a rate of 250 rpm within a water bath maintained at a temperature of 20 °C. Samples were collected at certain time intervals of 5, 10, 15, 30, 60, 120, 240, 360, 480, and 1440 min in order to conduct tests on the levels of boron and arsenic.

Pseudo first- and second-order kinetic models were employed to assess the collected kinetic data (Equations (1) and (2)) [29].

(1) $$log\left(q_{e}–q_{t}\right)=logq_{e}–\frac{k_{1}}{2.303}t$$

(2) $$\frac{t}{q_{t}}=\frac{1}{k_{2}q_{e}^{2}}+\frac{1}{q_{e}}$$

where, *q_e_* represents the quantity of the substance that is adsorbed at the state of equilibrium (mg/g); *q_t_* denotes the quantity of the substance that is adsorbed at any given time *t* (mg/g). The variables *k*_1_ and *k*_2_ represent the rate constants for adsorption in a pseudo 1st order and pseudo 2nd order reaction, respectively. The unit for *k*_1_ is min^−1^, whereas *k*_2_ is expressed in g/mg min.

#### 3.2.3. Batch-Mode Elution Tests

For analyzing the elution performance of boron from the chelating resins, sorption of boron with the 1JW, 2JW, and Diaion CRB 05 resins was carried out with both model boric acid solution (MBAS) (10 mg B/L) and geothermal water by using optimum resin concentrations. For the sorption step, the chelating resins were put in contact with 25 mL of MBAS or geothermal water at 25 °C for 24 h in a shaker. The ideal resin concentrations used in elution studies are listed in Table 12.

Once the equilibrium was attained, the chelating resins underwent filtration and were then rinsed with ultrapure water. The boron-loaded resins were subjected to contact with 25 mL of 0.5 M H_2_SO_4_. The elution process was conducted using a water bath at a temperature of 25 °C, accompanied by a shaker operating at a speed of 70 rpm. The elution process consisted of three consecutive phases, each having an exposure period of 2 h, 2 h, and 1 h, consecutively. On top of that, the elution process was also conducted using a 1 M H_2_SO_4_ solution in a similar manner. The concentrations of boron after sorption and elution were measured, and the amount of boron eluted was determined. Percent elution (*E*) was calculated with Equation (3).

(3) $$\;E\;\left(\%\right)=\frac{C_{el}\times V_{el}}{\left(C_{0}–C_{t}\right)\times V_{ads}}\times 100\;$$

where, *C_o_* is the concentration of boron in the initial solution for sorption (mg/L), *C_t_* is the concentration of boron remaining in the solution after sorption (mg/L), *C_el_* is the concentration of boron in the eluate (mg/L), *V_ads_* is the volume of solution in the sorption step (L), and *V_el_* is volume of the elution solution (L).

#### 3.2.4. Column-Mode Sorption and Elution Tests

Column-mode sorption and elution studies were carried out using boron selective chelating resins 1JW, 2JW and Diaion CRB05. The column setup consists of a glass column featuring an interior diameter of 0.7 cm, a peristaltic pump to provide a certain feed flow rate, and a fraction collector to collect samples periodically. Before the column study, the resins were kept swollen in the ultrapure water for one day, and 0.5 mL of the swollen resin was measured and placed into the glass column. In the column mode sorption studies, geothermal water was used as the feeding solution. During the sorption stage, 3 mL (6 BV, BV: bed volume) samples were collected at a space velocity of 20/h. When the concentration of boron in the effluent samples collected by the fraction collector equaled the concentration of boron in the feed, the column was stripped with a 5% H_2_SO_4_ solution after being rinsed with ultrapure water. During the stripping stage, 2 mL (4 BV) samples were collected at a space velocity of 10/h. Ultrapure water was passed through the column again after stripping. Breakthrough curves of boron in accordance with the analysis were obtained for each resin. Breakthrough ($q_{B}$) and total capacities ($q_{Eq}$) of resin samples were calculated using Equations (4) and (5). Following the sorption studies, elution studies were also carried out, and the total elution ($m_{Elution}$) of the resin was calculated by using Equation (6), while the elution efficiency ($E_{eff}$) was obtained by using Equation (7).

(4) $$q_{B}=\frac{\int_{0}^{V_{B}}\left(C_{o}-C\right)\;dV}{m_{resin}}\;$$

(5) $$q_{Eq}=\frac{\int_{0}^{V_{Eq}}\left(C_{o}-C\right)\;dV}{m_{resin}}\;$$

(6) $$m_{Elution}=\int_{0}^{{V_{E}}_{final}}C_{E}\;dV\;$$

(7) $$E_{eff}\left(\%\right)=\frac{\;Total\;elution\;of\;the\;resin}{Total\;capacity\;of\;the\;resin}\times 100\;$$

where, *C_o_* is the initial concentration of the boron in the feed solution (mg/L), *C* is the concentration of boron in the effluent collected after sorption (mg/L), and *C_E_* is concentration of boron in the eluate fraction (mg/L). Also, *V_B_*, and *V_Eq_* are the total volume of the sorption solution at the breakthrough point and at the equilibrium (L), respectively, while *V_Efinal_* is the volume of the elution solution (L).

#### 3.2.5. Analysis

The spectrophotometric curcumin method was performed to analyze the boron concentration by using the Jasco V-530 spectrophotometer (Jasco Inc., Easton, MD, USA) at λmax 543 nm. The arsenic concentration was measured by using ICP-MS (Agilent Technologies 7900, Agilent Technologies, Inc., Santa Clara, CA, USA). The Hach Lange HQ14D model multimeter was used for pH, conductivity, TDS, and salinity measurements, while the Hach Lange DR3900 model spectrophotometer was employed for measuring the silica concentration by utilizing Silica 3 Reagent Powder Pillows. A Shimadzu AA-7000 model atomic absorption spectrophotometer was used for cations analyses, and a Shimadzu Prominence HIC-SP model ion chromatography device was used for anions analyses. Bicarbonate concentrations were determined by using the titrimetric method.

## 4. Conclusions

In the sorption studies with geothermal water, the novel gel-like 1JW and 2JW resins produced competitive results compared with Diaion CRB05 in the case of boron removal. The regeneration of resins was more efficient for both the 1JW and 2JW resins, despite their gel-like structures, while the boron selective commercial chelating resin was porous. Additionally, the 1JW and 2JW resins showed much better sorption of arsenic than their commercial analogue. The synthesized sorbents show potential for the concurrent removal of boron and arsenic from geothermal water.

## Figures and Tables

**Table 1:** The concentration of boron remaining in the solution after sorption.

Amount of Adsorbent (g Resin/L Solution)	The Concentration of Boron in the Solution after Sorption (mg/L)		
	1JW	2JW	Diaion CRB05
0	10.06	10.06	10.06
2	0.87	0.13	1.97
4	0.26	0.03	0.18
8	0.07	0.03	0.06
16	0.03	0.03	0.05
32	0.03	0.02	0.05

**Table 2:** The concentration of arsenic remaining in the solution after sorption.

Amount of Adsorbent (g Resin/L Solution)	The Concentration of Arsenic in the Solution after Sorption (mg/L)		
	1JW	2JW	Diaion CRB05
0	0.160	0.160	0.160
2	0.075	0.070	0.121
4	0.026	0.019	0.069
8	0.008	0.009	0.040
16	0.006	0.006	0.020
32	0.004	0.006	0.009

**Table 3:** Comparison of boron and arsenic removal with various chelating resins.

Resin	Feed Boron Concentration (mg/L)	Resin Amount (g/L)	Removal (%)	Reference
1JW	10.06	2	91.0	This Study
2JW	10.06	2	98.6	This Study
CRB05	10.06	4	98.7	This Study
1PTN	10.90	32	64.0	[23]
2PTN	10.90	16	96.0	[23]
poly(GMA-co-EDM) containing NMDG	11.00	4	94.0	[24]
37Diaion CRB05	4.43	2	90.0	[25]
Diaion CRB02	10.50	3.2	85.0	[26]
P(VbNMDG)	10.50	1.6	98.0	[26]
**Resin**	**Feed Arsenic Concentration (mg/L)**	**Resin Amount** **(g/L)**	**Removal (%)**	
1JW	0.160	8	94.8	This Study
2JW	0.160	8	94.1	This Study
Diaion CRB05	0.160	32	94.0	This Study
1PTN	0.160	32	18.0	[23]
2PTN	0.160	16	93.0	[27]
PVBNMDG	10.0	5	95.0	[21]
NMDG-DMA	1.00	1.6	95.2	[22]

**Table 4:** The concentration of boron remaining in the solution after adsorption.

Time (min)	The Concentration of Boron in the Solution after Sorption (mg/L)		
	1JW	2JW	Diaion CRB05
0	8.4	9.5	9.9
5	5.2	4.2	7.2
10	4.6	4.1	0.6
15	3.6	3.3	0.5
30	2.3	2.1	0.5
60	1.6	1.2	0.2
120	0.6	1.0	0.1
240	0.5	0.4	0.1
360	0.2	0.3	0.1
480	0.7	0.3	0.1
1440	1.1	0.2	0.1

**Table 5:** Equations of diffusional and reactional kinetic models.

Model *	Equation **	Rate Determination Step
ISV	F(X) = −ln(1 − X) = K_1i_ where K_1i_ = 3DC/r_o_δC_r_	Film Diffusion
ISV	F(X) = −ln(1 − X^2^) = kt where k = D_r_π^2^/r_o_^2^	Particle Diffusion
UC	F(X) = X = (3C_Ao_K_mA_/a_ro_C_so_)t	Liquid Film
UC	F(X) = 3−3(1 − X)^2/3^-2X = (6D_eR_C_Ao_/a_ro_^2^C_so_)t	Reacted Layer
UC	F(X) = 1− (1 − X)^1/3^ = (k_s_C_Ao_/a_ro_C_o_)t	Chemical Reaction

* ISV: Infinite Solution Volume, UCM: Unreacted Core; ** X = *q_t_*/*q_e_*_._

**Table 6:** R^2^ values calculated for each resin using conventional and diffusional/reaction models.

Resins	Conventional Kinetic Modeling		Diffusional and Reaction Models				
			Infinite Solution Volume		Unreacted Core Model		
	Pseudo 1st Order	Pseudo 2nd Order	Film Diffusion	Particle Diffusion	Liquid Film	Reacted Layer	Chemical Reaction
1JW	R^2^: 0.98	R^2^: 0.99	R^2^: 0.96	R^2^: 0.99	R^2^: 0.64	R^2^: 0.92	R^2^: 0.98
	q_e_: 0.97	q_e_: 0.19					
	k_1_: 0.41	k_2_: 55.2					
2JW	R^2^: 0.81	R^2^: 0.99	R^2^: 0.96	R^2^: 0.99	R^2^: 0.46	R^2^: 0.70	R^2^: 0.82
	q_e_: 0.98	q_e_: 0.83					
	k_1_: 0.80	k_2_: 6.55					
Diaion CRB 05	R^2^: 0.80	R^2^: 0.99	R^2^: 0.96	R^2^: 0.99	R^2^: 0.95	R^2^: 0.78	R^2^: 0.85
	q_e_: 0.59	q_e_: 0.68					
	k_1_: 1.62	k_2_: 5.32					

q_e_ (mg/g); k_1_ (1/min).

**Table 7:** Boron elution data.

			SORPTION		ELUTION	
Sorption Feed	Eluent	Resin	m_aft_s_ (mg)	Boron Removal (%)	m_aft_el_ (mg)	Elution Efficiency (%)
MBAS	0.5 M H_2_SO_4_	1JW	0.02	91.7	0.18	74.1
		2JW	0.02	91.7	0.23	93.6
		CRB 05	0.03	89.7	0.24	99.0
	1 M H_2_SO_4_	1JW	0.03	89.2	0.23	99.5
		2JW	0.02	90.6	0.22	96.8
		CRB 05	0.02	91.3	0.23	99.0
GW	1 M H_2_SO_4_	1JW	0.04	83.38	0.21	97.7
		2JW	0.01	94.29	0.20	99.8
		CRB 05	0.02	93.30	0.24	99.8

MBAS: Model boric acid solution; GW: Geothermal water; m_aft_s_: The amount of boron that remains in the solution after sorption. m_aft_el_: The amount of boron in the solution after elution.

**Table 8:** Overall results of column-mode studies.

The Data Calculated from the Breakthrough Curves	1JW	2JW	Diaion CRB05
Breakthrough capacity (mg B)	1.76	0.91	1.99
The breakthrough capacity of resin (mg/mL resin)	3.52	1.82	3.98
Total capacity (mg B)	2.54	2.32	3.15
The total exchange capacity of resin (mg B/mL resin)	5.08	4.64	6.29
Degree of column utilization (%)	69.3	39.4	63.1
Total eluted boron (mg B)	1.86	2.22	2.85
Elution efficiency (%)	73.1	95.7	90.4

**Table 9:** The characteristics of gel-type 1JW and 2JW resins.

Parameters	1JW	2JW
Resin type	Gel	Expanded gel
Composition	VBC/S/DVB	VBC/DVB
Water content (%)	42	78
IEC (mmol/g)	2.1	2.3
Functional group	(R-N(CH_3_)-C_6_H_8_(OH)_5_)	

VBC: vinylbenzyl chloride; S: styrene; DVB: divinyl benzene (crosslinking agent); IEC: ion exchange capacity.

**Table 10:** The characteristics of Diaion CRB 05 (Mitsubishi Chem., Tokyo, Japan).

Parameters	Diaion CRB 05
Resin type	Chelating resin
Composition	S/DVB
Water content (%)	43–53
IEC (mmol/g)	0.95
Functional group	(R-N(CH_3_)-C_6_H_8_(OH)_5_)

**Table 11:** The chemical composition of geothermal water.

Specifications	GW	Specifications	GW
pH	8.40	Ca^2+^ (mg/L)	29.76
EC (µS/cm)	1721	Mg^2+^ (mg/L)	7.11
TDS (mg/L)	863	F^−^ (mg/L)	6.34
Salinity (‰)	0.87	Cl^−^ (mg/L)	209.67
(HCO_3_)^−^ (mg/L)	655	NO_3_^−^ (mg/L)	2.22
Li^+^ (mg/L)	1.24	SO_4_^2−^ (mg/L)	161.6
Na^+^ (mg/L)	309.6	B (mg/L)	10.94
K^+^ (mg/L)	27.9	SiO_2_ (mg/L)	118.9
NH_4_^+^ (mg/L)	2.94	As (mg/L)	0.160

**Table 12:** The amounts of chelating resins employed in the batch elution procedures.

Resin	1JW	2JW	Diaion CRB 05
Resin amount (g resin/L solution)	4.00	4.00	2.00

## Data Availability

The data available in this study are available on request from the corresponding author.

## References

[1] Abu-Zeid M., Shiklomanov I.A. (2003). Water Resources as a Challenge of the Twenty-First Century. Tenth IMO Lecture.

[2] Shiklomanov I.A. (1993). Water in Crisis: A Guide to the World’s Fresh Water Resources.

[3] Jhansi S.C., Mishra S.K. (2013). Wastewater Treatment and Reuse: Sustainability Options. Consilience.

[4] U.S. Environmental Protection Agency Basic Information about Water Reuse. https://www.epa.gov/waterreuse/basic-information-about-water-reuse.

[5] Englande A.J., Krenkel P., Shamas J. (2015). Wastewater Treatment & Water Reclamation. Ref. Modul. Earth Syst. Environ. Sci..

[6] Baba A. (2015). Application of Geothermal Energy and Its Environmental Problems in Turkey. Int. J. Glob. Environ. Issues.

[7] Richter A. (2016). Mapping the Icelandic Geothermal Energy Sector.

[8] Tomaszewska B., Rajca M., Kmiecik E., Bodzek M., Bujakowski W., Wątor K., Tyszer M. (2017). The Influence of Selected Factors on the Effectiveness of Pre-Treatment of Geothermal Water during the Nanofiltration Process. Desalination.

[9] Melikoglu M. (2017). Geothermal Energy in Turkey and around the World: A Review of the Literature and an Analysis Based on Turkey’s Vision 2023 Energy Targets. Renew. Sustain. Energy Rev..

[10] Bundschuh J., Tomaszewska B. (2018). Geothermal Water Management.

[11] Shah M., Sircar A., Varsada R., Vaishnani S., Savaliya U., Faldu M., Vaidya D., Bhattacharya P. (2019). Assessment of Geothermal Water Quality for Industrial and Irrigation Purposes in the Unai Geothermal Field, Gujarat, India. Groundw. Sustain. Dev..

[12] Yoshizuka K., Kabay N., Bryjak M. (2010). Arsenic and Boron in Geothermal Water and Their Removal. The Global Arsenic Problem.

[13] Bundschuh J., Maity J.P., Nath B., Baba A., Gunduz O., Kulp T.R., Jean J.-S., Kar S., Yang H.-J., Tseng Y.-J. (2013). Naturally Occurring Arsenic in Terrestrial Geothermal Systems of Western Anatolia, Turkey: Potential Role in Contamination of Freshwater Resources. J. Hazard. Mater..

[14] Bryjak M., Wolska J., Soroko I., Kabay N. (2009). Adsorption-Membrane Filtration Process in Boron Removal from First Stage Seawater RO Permeate. Desalination.

[15] Yoshizuka K., Nishihama S. (2015). Separation and Recovery of Boron From Various Resources Using Chelate Adsorbents. Boron Separation Processes.

[16] Belova T.P., Ershova L.S. (2021). Boron Concentration by Industrial Anion Exchanger Resins from Model Solutions in a Dynamic Mode. Heliyon.

[17] Figueira M., Reig M., Fernández de Labastida M., Cortina J.L., Valderrama C. (2022). Boron Recovery from Desalination Seawater Brines by Selective Ion Exchange Resins. J. Environ. Manag..

[18] Hoang V.A., Nishihama S., Yoshizuka K. (2019). Adsorptive Removal of Arsenic from Aqueous Environment. J. Chem. Eng. Jpn..

[19] Xia S., Dong B., Zhang Q., Xu B., Gao N., Causseranda C. (2007). Study of Arsenic Removal by Nanofiltration and Its Application in China. Desalination.

[20] Akin I., Arslan G., Tor A., Cengeloglu Y., Ersoz M. (2011). Removal of Arsenate [As(V)] and Arsenite [As(III)] from Water by SWHR and BW-30 Reverse Osmosis. Desalination.

[21] Urbano B.F., Rivas B.L., Martinez F., Alexandratos S.D. (2012). Water-Insoluble Polymer-Clay Nanocomposite Ion Exchange Resin Based on *N*-Methyl-d-Glucamine Ligand Groups for Arsenic Removal. React. Funct. Polym..

[22] Dambies L., Salinaro R., Alexandratos S.D. (2004). Immobilized N-Methyl-d-Glucamine as an Arsenate-Selective Resin. Environ. Sci. Technol..

[23] Cyganowski P., Şen F., Altıok E., Wolska J., Bryjak M., Kabay N., Arda M., Yüksel M. (2021). Surface-Activated Chelating Resins Containing *N*-Methyl-d-Glucamine Functional Groups for Desalination of Geothermal Water Aimed for Removal of Boron and Arsenic. Solvent Extr. Ion Exch..

[24] Samatya S., Tuncel A., Kabay N. (2012). Boron removal from geothermal water by a novel monodisperse porous poly (GMA-co-EDM) resin containing *N*-methyl-d-glucamine functional group. Solvent Extr. Ion Exch..

[25] Ozkula G., Urbano B.F., Rivas B.L., Kabay N., Bryjak M. (2016). Arsenic sorption using mixtures of ion Exchange resins containing N-methyl-D-glucamine and quaternary ammonium groups. J. Chil. Chem. Soc..

[26] Santander P., Rivas B.L., Urbano B.F., İpek İ.Y., Özkula G., Arda M., Yüksel M., Bryjak M., Kozlecki T., Kabay N. (2013). Removal of boron from geothermal water by a novel boron selective resin. Desalination.

[27] Wolska J., Bryjak M. (2011). Preparation of Polymeric Microspheres for Removal of Boron by Means of Sorption-Membrane Filtration Hybrid. Desalination.

[28] Ibezim-Ezeani M.U., Okoye F.A., Akaranta O. (2012). Kinetic Studies on the Removal of Some Metal Ions from Aqueous Solution Using Modified Orange Mesocarp Extract. Int. J. Water Resour. Environ. Eng..

[29] Hameed B.H., Rahman A.A. (2008). Removal of Phenol from Aqueous Solutions by Adsorption onto Activated Carbon Prepared from Biomass Material. J. Hazard. Mater..

